# Data Sharing in Neurosurgery and Neurology Journals

**DOI:** 10.7759/cureus.2680

**Published:** 2018-05-23

**Authors:** Jeremiah N Johnson, Keith A Hanson, Caleb A Jones, Ramesh Grandhi, Jaime Guerrero, Jesse S Rodriguez

**Affiliations:** 1 Department of Neurosurgery, Baylor College of Medicine, Houston, USA; 2 School of Medicine, University of Texas Health Science Center San Antonio, San Antonio, USA; 3 Department of Neurological Surgery, University of Texas Health Science Center San Antonio, San Antonio, USA

**Keywords:** data sharing, dark data, neuroscience, databases, journal policy, neurosurgery, neurology, reproducibility, open science

## Abstract

In this era of high health care cost and limited research resources, open access to de-identified clinical research study data may promote increased scientific transparency and rigor, allow for the combination and re-analysis of similar data sets, and decrease un-necessary replication of unpublished negative studies. Driven by expanded computing capabilities, advocacy for data sharing to maximize research value is growing in both translational and clinical research communities. The focus of this study is to report on the current status of publicly available research data from studies published in the top 40 neurology and neurosurgery clinical research journals by impact factor. The top journals were carefully reviewed for data sharing policies. Of the journals with data sharing policies, the 10 most current original research papers from December 2015 – February 2016 were reviewed for data sharing statements and data availability. A data sharing policy existed for 48% (19/40) of the 40 journals investigated. Of the 19 journals with an existing data sharing policy, 58% (11/19) of the policies stated that data should be made available to interested parties upon request and 21% (4/19) of these journals encouraged authors to provide a data sharing statement in the article of what data would be available upon request. Of the 190 articles reviewed for data availability, 21% (40/190) of these articles included some source data in the results, figures, or supplementary sections. This evaluation highlights opportunities for neurology and neurosurgery investigators and journals to improve access to study data and even publish the data prospectively for the betterment of clinical outcome analysis and patient care.

## Introduction

**Figure 1:**
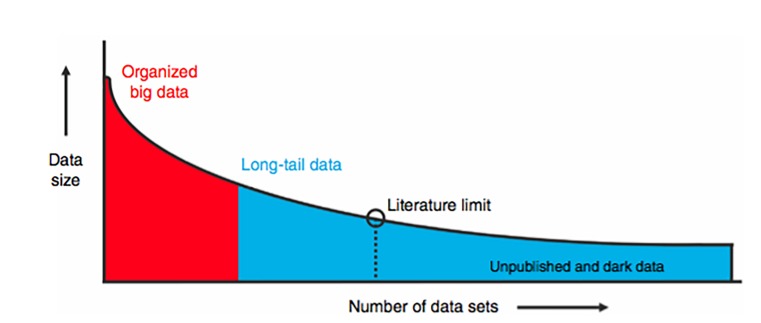
Schematic Illustration of Long Tail Data “Studies that have plotted data set size against the number of data sources reliably uncover a skewed distribution. Well-organized big science efforts featuring homogenous, well-organized data represent only a small proportion of the total data collected by scientists. A very large proportion of scientific data falls in the long-tail of the distribution, with numerous small independent research efforts yielding a rich variety of specialty research data sets. The extreme right portion of the long tail includes data that are unpublished; such as siloed databases, null findings, laboratory notes, animal care records, etc. These dark data hold a potential wealth of knowledge but are often inaccessible to the outside world” [1]. Image courtesy of Nature Publishing Group.

The concept that scientists and clinicians may benefit from sharing primary research data has been around since the early days of the Internet [1]. Open data sharing has roots in promoting study transparency, reproducibility, and decreasing waste [2-4]. Neuroscience, like many fields, suffers from biases and inaccuracies introduced by small sample sizes, which can lead to bias and difficulty with reproducibility [5, 6]. For example, recent attempts to reproduce classic studies in the fields of psychology and cancer biology have yielded reproducibly rates of 40% and 10%, respectively [7, 8]. Arguments for making primary study data available is that it allows for data pooling of small size data sets, data re-analysis by independent investigators, and facilitation of cooperative research efforts to build sample size. No scientific study is safe from experimental error and/or methodological limitations, and more open access to primary data may promote increased scientific vigilance due to the publishing investigators' knowledge of the potential for the data to be independently re-analyzed and its validity tested. The use of shared data also constitutes a form of open peer review, as it is in the best interest of the second set of investigators to confirm the quality of the data and validate the methodology used to obtain it. Clinical research lends itself to numerous small-scale studies published in a myriad of journals, and although the individual data sets may be small, when combined they represent a large proportion of overall clinical data available. These data have been termed long tail data, and studies collecting long tail data compose the major component of scientific funding [1,9] (Figure 1). An additional issue at play is the estimated 50% of completed studies in medicine that go unreported because findings are not in line with author hypothesis and / or negative findings [10]. Failure to disseminate and publish unreported “dark data” (negative results) can result in publication bias, needless duplication of scientific studies, and contributes to failures in scientific replication [1, 2, 6] .

The utilization of dark and long-tail data is of particular importance in neuroscience and neurosurgery research, as publications in these fields commonly suffer from small sample sizes resulting in low study power and poor reproducibility [5]. Larger data set analysis can lead to identification of rare events and may identify previously undetected statistical trends to help guide the direction of future research efforts. Given the prevalence of neurological disorders and the substantial public resources that have been dedicated to the discovery of new therapeutics, great advances should be expected if researchers were to efficiently utilize and share source data in neurosurgery. Data sharing has recently become a point of emphasis at the National Institute of Health as well as guidelines enacted by the International Committee of Medical Journal Editors. To date, there has not been an empirical evaluation of publicly available primary data and related material and protocols in the clinical neuroscience journals. In this report, we aim to assess the current status of these practices in the most highly cited neurosurgery and neurology-related clinical journals. This empirical evaluation highlights opportunities for improvement, to fully engage all investigators to contribute and practice data sharing, to better utilize scientific resources, and to improve research data for the betterment of patient outcomes.

## Materials and methods

To comprise the list of the most clinically relevant journals pertaining to neurology and neurosurgery, per the investigator’s experience, a 2014 Institute of Scientific Information (ISI) Web of Knowledge Journal Citation Report was generated. The journal subject category search terms used were Clinical Neurology, Neuroimaging, and Neurosciences. The internet address for the search is provided; http://admin-apps.webofknowledge.com/JCR/JCR?RQ=LIST_SUMMARY_JOURNAL&cursor =1. The Web of Knowledge citation reports are now maintained by Clarivate Analytics. Journals were sorted by 2014 impact factor (highest to lowest). The top 40 clinical journals by impact factor and relevancy were selected for further investigation to determine if each journal had an existing data sharing policy (Table 1).

**Table 1:** Top 40 Journals by Impact Factor as Generated from the ISI Journal of Knowledge Website Citation Report in 2014 This list represents all the journals whose policies and articles were reviewed for this paper.

Journal Title	2014 Impact Factor	Data Sharing Policy?
Lancet Neurology	21.896	Y
Annals of Neurology	9.977	Y
Neurology	8.185	Y
JAMA Neurology	7.271	N
Journal of Neurology, Neurosurgery, and Psychiatry	6.807	Y
Neuro-Oncology	6.776	N
Stroke	5.761	Y
Movement Disorders Journal	5.68	N
Journal of Cerebral Blood Flow and Metabolism	5.407	Y
Cephalalgia	4.891	Y
Multiple Sclerosis Journal	4.822	Y
Epilepsia	4.571	N
European Journal of Neurology	4.055	N
Cerebrovascular Diseases	3.754	N
Journal of Neurosurgery	3.737	Y
Journal of Neurotrauma	3.714	N
Neurosurgery	3.62	N
Journal of Neurology	3.377	N
Journal of Neuro-Oncology	3.07	N
World Neurosurgery	2.878	N
Journal of Headache and Pain	2.801	Y
Journal of Neurointerventional Surgery	2.774	Y
Headache	2.758	Y
European Journal of Vascular and Endovascular Surgery	2.49	Y
The Spine Journal	2.426	N
Journal of Neurosurgery: Spine	2.383	Y
Spine	2.297	N
Epilepsy and Behavior	2.257	Y
Current Neurovascular Research	2.253	N
Clinical Neuroradiology	2.25	N
European Spine Journal	2.066	N
Stereotactic and Functional Neurosurgery	2.019	N
Epilepsy Research	2.015	N
Parkinson's Disease	2.01	N
Spinal Cord	1.804	Y
ACTA Neurochirurgica	1.766	Y
Journal of Child Neurology	1.717	Y
Journal of Clinical Neurology	1.7	N
Journal of Stroke and Cerebrovascular Diseases	1.669	N
Journal of Neurosurgery: Pediatrics	1.482	Y

Each journal’s website was searched for relevant data sharing policies. All journal sections were studied to determine the availability of a data sharing policy, including the information about the journal, instructions for authors, and the editorial/publisher information. For each journal, the policies related to public availability and sharing of data were documented where available (accessed January 2016). Each paper was reviewed by going through the text, supplementary material, and the links available on the online version. The investigators also considered whether the paper was based on data covered by a journal policy. Data extraction from the 190 papers was performed, and the availability of source data, defined here as unprocessed data presented prior to statistical analysis, was recorded. When a data sharing policy could not be located for a particular journal, an email was sent to an editorial board member or the designated journal contact for further inquiry and responses were recorded.

## Results

**Figure 2:**
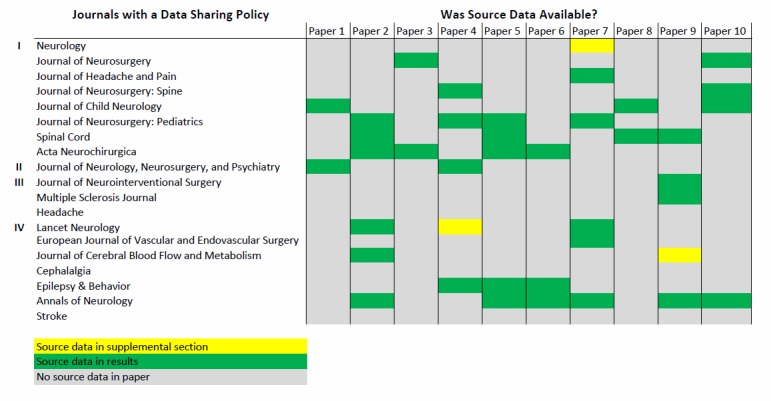
Data Sharing Policy and Source Data Availability by Journal I - Policy stated that data was to be made available on request II - Policy encouraged a statement from the author in the paper regarding what data is available III - Both I and II IV - Other

**Figure 3:**
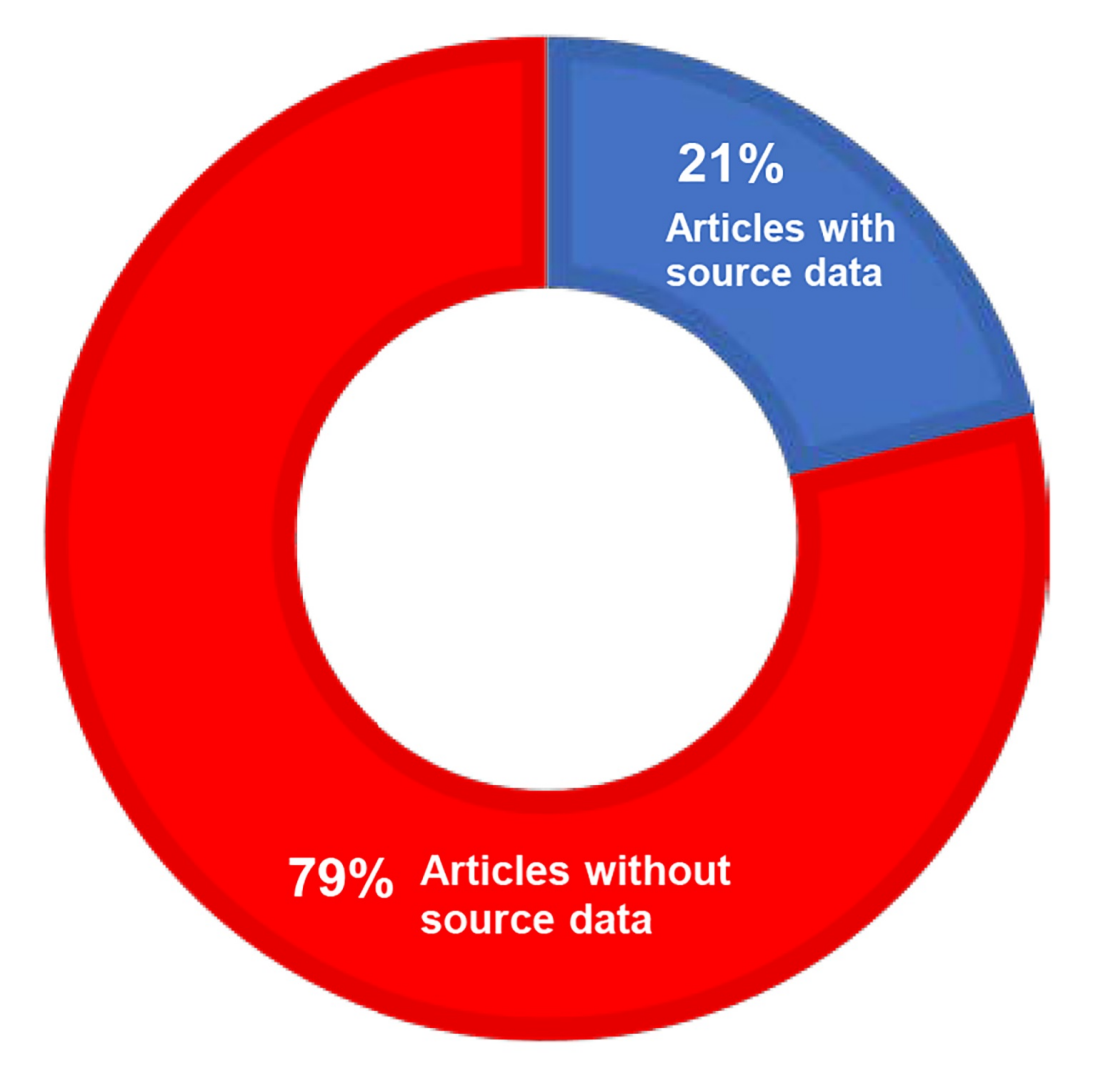
Source Data Availability for the 190 Reviewed Journal Articles

Over 380 neurology, neuroscience, and neurosurgery related journals were produced by the 2014 ISI Journal Citation Report query. A data sharing policy existed for 48% (19/40) of the 40 journals investigated (Table 1). Of the 19 journals with a data sharing policy, 58% (11/19) stated that data should be made available to interested parties upon request, and 21% (4/19) of these journals encouraged authors to provide a data sharing statement in the article of what data would be available upon request (Figure 2). Of the 190 papers reviewed for data availability, 21% (40/190) of these articles included some source data in the results, figures, or supplementary sections (Figure 3).

Subset analyses was performed of the 10 most recent published articles (range October 2015 – February 2016) from the top five journals by impact factor to detail variables associated with the types of studies published, the institute country of origin, and funding source. Each journal and article were assigned an alphanumeric identifier, labeled alphanumerically in descending order by impact factor and sequentially by article, e.g., A1-10, B1-10, etc. Representation of the 10 articles for journal A revealed that randomized controlled trials constituted 60% of the publications, 40% were funded by the National Institutes of Health (NIH), 40% were institutions based in the USA and 60% European, 20% reported some source data (A2 and A7) and all reported summary (averaged) and supplemental data. Journal A does state that all source data may be required to be accessible for 10 years after publication; however, the recipient of such data is more likely the publisher and not scientific investigators. Results of the 10 articles for Journal B revealed that randomized controlled trials constituted 10% of the publications with a majority of the publications being retro- or prospective observation studies, 20% were NIH funded and the majority were funded by foreign states or pharmaceutical industry, 30% were USA/Canadian institutions and 70% of international origin, 60% reported some source data (B2, B5-7, B9 and B10) and all reported summary (averaged) and supplemental data. Journal B does require authors to make their data available to other investigators for the purpose of verifying and replicating results. However, none of the articles in this journal mentioned such options to readers.

Analysis of the 10 articles from Journal C revealed that randomized controlled trials constituted 0% of the publications with a majority being retro- or prospective observational studies. None were NIH funded, 100% were European or Canadian studies, and none reported some source data and all reported summary (averaged) and supplemental data. While Journal C does not specifically require that source data be provided at submission, it does require it to be available upon request. None of the articles surveyed mentioned this option anywhere in the text. Analysis of the 10 articles from Journal D revealed that randomized controlled trials constituted 0% of the publications with a majority being retro- or prospective observational studies, estimated 50% state funded and 50% pharmaceutical funded. 10% of the studies were from USA-based institutions and 90% were from European or Asian countries, and 20% reported some source data while all reported summary (averaged) and supplemental data. This journal (D) specifically required a data sharing statement, for which only 30% of the publications included such a statement. Analysis results of the 10 articles for Journal E revealed that all studies were retro- or prospective studies, 40% were NIH funded, 60% were from USA institutions, 20% reported some source data (E2 and E3), and all reported summary (averaged) and supplemental data. Journal E specifically stated, “The American Heart Association (AHA) requires grant applicants to include a data sharing plan as part of the application process. Any research data that is needed for independent verification of research results must be made freely and publicly available within 12 months of the end of the funding period (and any no-cost extension).” However, the AHA did not fund any research described in the articles surveyed from Journal E.

## Discussion

The purpose of this analysis was to document the availability of source data in neurosurgery-related journals. These results demonstrate that, of the top 40 journals by impact factor in the discipline of neurosurgery and related fields, approximately half of the journals have an existing data sharing policy. Of the journals with an existing policy, approximately 60% included in the data sharing policy that data should be made available to interested parties upon request. Only 20% of these journals encouraged the authors to make this statement. Further, only 21% of the 190 articles reviewed included some source data in the results section or in the supplemental information. Results from the top 5 journals (10 sequential research articles evaluated from each) show that 20% of the articles included some source data with a majority of the studies being retrospective or prospective in nature. There was no clear pattern of funding source or country of study origination in the journal articles surveyed. Overall, our findings demonstrate a lack of emphasis on data sharing among the top clinical neurology and neurosurgery journals with less than half of the journals having data sharing policies. Furthermore, even among the journals with data sharing policies, we found an inconsistent application of the policies by investigators publishing in the journals, which is consistent with other empiric analyses of data sharing in the literature [11, 12].

Data sharing in neuroscience was initially driven by the computational neuroscience and neuroimaging community that sought data integration for brain function modeling. Over time, more groups have joined in emphasizing the benefits of creating large aggregated data sets not only for statistical purposes but also for reasons of transparency, reproducibility and minimizing waste [1]. The potential barriers to data sharing in clinical science are numerous and varied. From the perspective of investigators, reasons for avoiding sharing of data include industry funding, perception of data ownership, lack of recognition to the benefits of data sharing, cost and time to share, and concern about misuse of data [13-15]. To the author’s knowledge as of this writing, no standardized metrics or norms exist to benefit the investigator for sharing their data in the neurosurgery or neurology literature. Academic journals are not the only source of inconsistency in data sharing policy, as funding agencies often differ on their requirements for data availability. Although strides are being made, such as with the NIH final rule on clinical trial data sharing or the International Committee of Medical Journal Editors declaration that prompt data sharing is a condition for review, lack of consistent data sharing policy exists amongst funding agencies as well. This lack of consistency between agencies could encourage noncompliance. While NIH funded studies are required to make their data available, none of the NIH funded studies reviewed in our sample mentioned this opportunity. Regarding the physical sharing of data, it is simple to have qualitative data readily available, which is commonly reported in descriptive results for most clinical studies. However, quantitative source data are far more challenging data sets to have available as viewed by the generator of those data. In light of these challenges, it is impractical to expect the investigators to bear the entire burden and costs of sharing their data, and the time and energy required to peer review source data sets, for uniformity and quality must also be considered.

**Figure 4:**
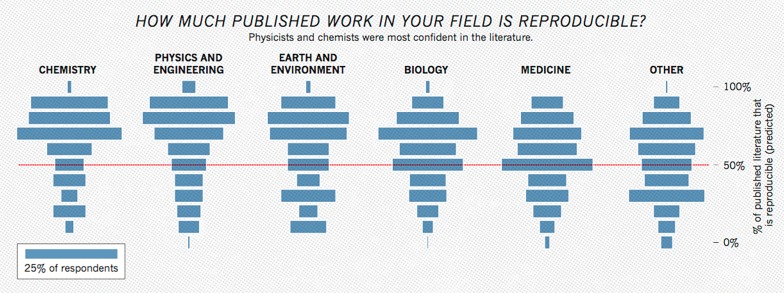
Plot Representing How Confident Researchers Were in the Reproducibility of Studies Within Their Field. Data is based on responses from a survey of 1,576 scientists through the journal Nature’s online website [17]. Image courtesy of Nature Publishing Group.

**Figure 5:**
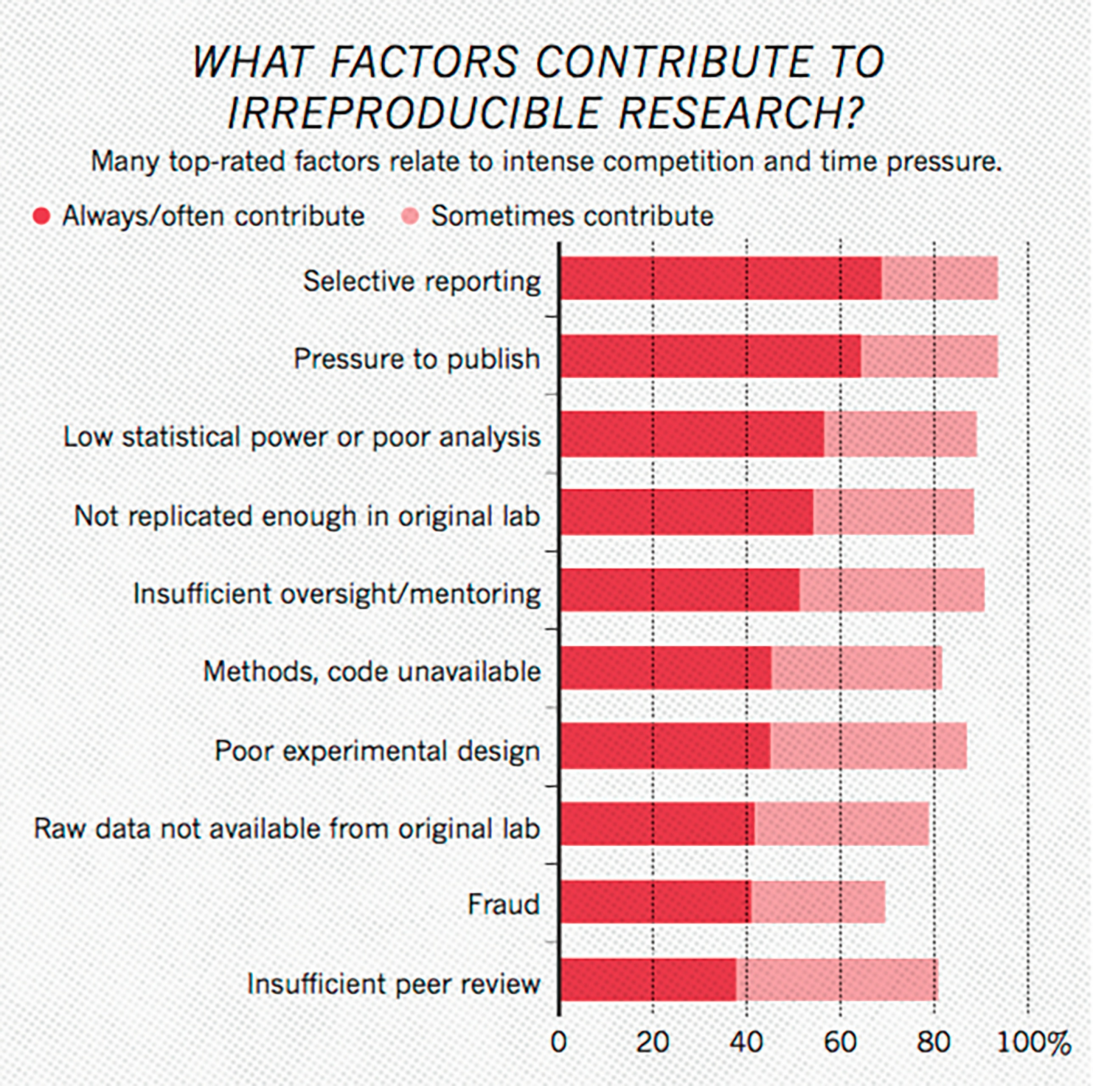
Plot Representing What Researchers Believe are the Primary Causes of Reproducibility Failure in the Sciences. Data is based on responses from a survey of 1,576 scientists through the journal Nature’s online website [17]. Image courtesy of Nature Publishing Group.

As numerous scientific disciplines grapple with an ongoing reproducibility crisis, open sharing of study data has been identified as a factor to improve transparency, re-analysis and boost reproducibility [7, 8, 16] (Figures 4, 5). Fortunately, there have already been some strides regarding how to motivate and expand data sharing, especially in fields that struggle to maintain adequate sample sizes. In the field of neuroscience, the NIH’s Brain Initiative as well as the Human Connectome Project have established interdisciplinary collections of neuroscience data. Such organizations allow for high quality data vetted by national organizations to be shared without additional effort from the original investigators. In addition, disease-specific repositories and data standards are being developed for conditions like polycystic kidney disease and, more relevant to this paper, spinal cord injury [17, 18]. Establishing disease or subfield-specific standards for sharing data would eliminate inconsistent data types and formats in the sharing of long-tail data. This, in turn, would allow for more expedient curation and analysis of this data with computer software. Pooling data in third party repositories is also beneficial to the longevity of the data, as there is some evidence to show that the availability of data decreases substantially over time when confined to the investigator [19]. Publishing data sets online to accompany the manuscript [20] or in separate independent scientific data journals [21, 22] and repositories [23] can allow peers to easily access scientific study data sets. Further work needs to be done in the areas of incentivizing investigators to publish data sets, organization of data set repositories into easily citable and discoverable formats [24], and policing the use and re-interpretation of published data [1]. Further, ever advancing machine learning and big data analytics algorithms may make even imperfectly standardized source data compliable and analyzable in the future. Another possible approach to data sharing in neurology and neurosurgery is reducing the accumulation of dark data through the use of prospective registries. Here data is collected, standardized, and added to an open national registry independent of any individual investigation. This data can then be used to analyze outcomes and the standard of care at a large scale. Establishing the rules of data reporting and ensuring that all collected data is open before it is collected would eliminate many of the barriers to data sharing discussed previously. Such use of registries has already had an impact on neurosurgery through the establishment of the Quality Outcomes Database (QOD), formerly known as the National Neurosurgery Quality Outcomes Database. This program has established registries for spinal cord injury, radiosurgery, cerebrovascular, as well as general outcomes data. The QOD has over 90 active centers contributing data, and the project has already made significant contributions to the literature, especially in spine [25-27].

In addition to the barriers to data sharing discussed above, another important consideration is how to efficiently manage and combine variable source data into common structured data as sharing becomes more commonplace. Prospective registries, community repositories, and institution-associated consortia are options to promote standardized sharing of data, but these approaches are limited. There will always be large quantities of novel, independent study data, and while we suggest that computer science may someday develop the power to overcome data inconsistencies on the processing side, it would be naïve to assume such advancements would correct all possible inconsistencies between data sources. Shared policies within the clinical neurosciences regarding how, when, and where data should be shared would be ideal. However, as discussed in Ferguson et al., a “one-size-fits-all” policy and infrastructure will likely be ineffective [1]. One proposed solution is to construct a universal indexing system where data types, wherever they are stored, are labeled according to source and form. Such a system has the benefit of allowing individual institutions and organizations to store data as they please while also allowing such data to be indexed by the scientific community at large. Another variable to consider within the data is varying outcome measures from study to study. Unless standardized outcome measures are established it will be difficult to draw confident conclusions from such data. Data sharing would have to be limited to studies that don’t involve subjective measures of outcome, or, more likely, would just have to be shared prior to the evaluation of any outcome. Primary source data as opposed to secondary outcomes data should be the gold standard in cases where strict outcomes guidelines don’t exist. One final concern regarding the data to be shared is copyright, intellectual property regulations, and institutional data transfer agreements. On this topic we feel that culture within the clinical neuroscience research community plays a very important role. A study by Blumenthal et al. suggests that while most investigators in the life sciences are willing to share data, the “most productive and entrepreneurial” among them were the most likely to withhold results for patent applications, etc. [28]. This observation, in addition to the well-known “publish or perish” mentality within the medical research community, suggests that the current system and culture of research rewards protective behavior with research data. This problem is compounded by the fact that research funding is often distributed disproportionately to the same high-profile investigators. The barriers that rampant copyright and intellectual property represent within the research community will be corrected when such methods are disincentivized. One possible way to correct this is to keep rewarding productivity, but have productivity measured, at least in part, on data production. An example of this would be to have academic impact resulting from data set publishing considered separate from manuscript publishing. This would allow data set citations to be included in academic impact calculations such as the h index [29]. A published manuscript reflects a moment in time that evaluates data based on present context, but the data will live on past the assumptions of its time. In that way it is more valuable and will likely give more to the community over time than a single paper. To summarize, some next steps needed to facilitate data sharing in the clinical neurosciences include the development of a data sharing infrastructure, adherence to a set of standard practices for formatting, publishing and citing data, incentivization of authors to format and publish their data freely, development of a culture of sharing, and finally funding models to power data repositories and searchability.

## Conclusions

The fields of neurosurgery and neurology are ever evolving with the development of novel approaches for the treatment of numerous disease states. Evidence based decisions in patient care are fundamental to treatment success and data sharing has been identified to be of importance for maximizing clinical neuroscience study outcome analysis, improving study reproducibility and promoting transparency while decreasing waste. Yet, our findings demonstrate a relative paucity of data sharing by neurology and neurosurgery clinical journals both in policy and in practice. Further awareness of possible improvement in data sharing in the clinical neuroscience literature is warranted.
